# Measuring In‐Shoe Plantar Strain Outcomes in the Presence of Callus: A Preliminary Study Using the STAMPS3D Measurement System

**DOI:** 10.1002/jfa2.70169

**Published:** 2026-06-13

**Authors:** Francesca Sairally, David A. Russell, Heidi J. Siddle, Daniele Trinca, Claire Brockett, Peter R. Culmer

**Affiliations:** ^1^ School of Mechanical Engineering Institute of Design, Robotics and Manufacturing University of Leeds Leeds UK; ^2^ Leeds Vascular Institute Leeds Teaching Hospitals NHS Trust Leeds UK; ^3^ Leeds Institute of Clinical Trials Research University of Leeds Leeds UK; ^4^ Leeds Institute of Rheumatic and Musculoskeletal Medicine University of Leeds Leeds UK; ^5^ Podiatry Department Leeds Teaching Hospitals NHS Trust Leeds UK; ^6^ NIHR Leeds Biomedical Research Centre Leeds UK; ^7^ School of Mechanical, Aerospace and Civil Engineering Insigneo Institute University of Sheffield Sheffield UK

**Keywords:** callus formation, diabetic foot, plantar pressure, plantar strain, shear stress, STAMPS3D

## Abstract

**Introduction:**

Diabetes‐related foot ulcers (DFU) are growing in prevalence, driven by the global rise in diabetes. Plantar callus has been identified as a risk factor for DFU formation, with its presence considered highly predictive for ulceration. Preventative strategies, such as debridement and offloading aim to reduce the thickness of localised hard skin formed through repetitive high pressure and shear stress. Although several studies have looked at the quantitative effects of callus and its removal in the neuropathic foot, assessments have relied solely on pressure measurements, neglecting the contribution of shear stress. A lack of in‐shoe measurement devices capable of measuring shear, either independently or alongside pressure, limits our understanding of how the foot behaves in the presence of callus or responds as a result of debridement.

**Methods:**

This proof‐of‐concept study investigates the use of the STAMPS3D system to capture cumulative 3D plantar strain data across the foot in the presence of simulated callus in healthy participants. By introducing multi‐layer silicone inserts that replicate key mechanical features of callus at the 1^st^ metatarsal head and heel regions, this study explores plantar strain responses in healthy participants.

**Results:**

The simulated callus produced subtle redistribution of strain across the foot with distinct local changes at the callus sites.

**Conclusion:**

Findings may inform future approaches to objective callus assessment, guide debridement practices and contribute to DFU prevention strategies.

## Graphical abstract

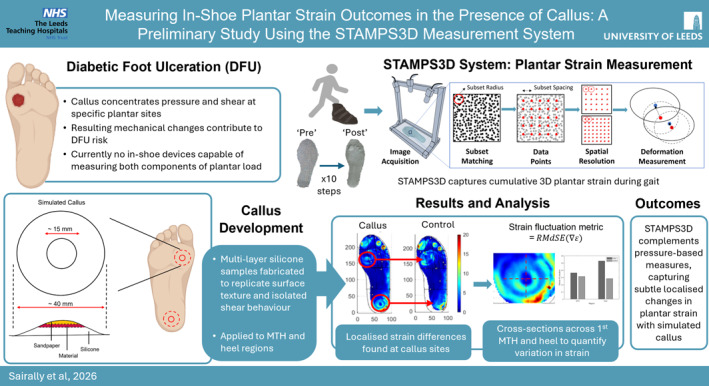

**Abbreviations**
DFU: Diabetic Foot Ulcer
DIC: Digital Image Correlation
IQR: Interquartile Range
MTH: Metatarsal Head
PPP: Peak Plantar Pressure
RMdSE: Root Median Squared Error
S_MAG_
: Magnitude of Strain
STAMPS: STrain Analysis and Mapping of the Plantar Surface

## 1 Introduction

Plantar callus is a dermatological response to repetitive high pressure and shear, resulting in localised thickening of the skin [1]. While often asymptomatic and protective in healthy individuals, callus can become pathological when pain develops or mechanical stress acts between callus, underlying soft tissue and prominent bone [2, 3]. In people with diabetes, and particularly peripheral neuropathy, this may go unnoticed, elevating the risk of tissue breakdown and ulcer formation. Accordingly, plantar callus is recognised as a risk factor and predictive marker for diabetic foot ulcers (DFUs) [2, 3, 4, 5, 6]. Callus debridement is a common intervention to reduce risk; in those with diabetic neuropathy studies report plantar pressure reductions by up to 30% after debridement [6, 7, 8] yet outcomes in other groups are mixed, with no significant change in rheumatoid arthritis [4, 9] or healthy cohorts [3]. These differences may suggest context‐dependent biomechanics influenced by pathology and sensory feedback.

Despite extensive literature linking callus to elevated plantar pressure, few studies have examined how callus and debridement impact plantar shear stress, an important contributor to plantar loading and skin breakdown. Increased peak‐to‐peak shear stress has been reported at callus sites in people with diabetes, even when plantar pressures do not differ between callused and non‐callused regions [10]. This suggests that shear may play a distinct role in callus formation and DFU risk.

Currently, no commercially available in‐shoe measurement devices can capture both pressure and shear, or shear alone, so plantar pressure remains a gold‐standard. Furthermore, existing technologies are typically time‐consuming, costly and complex to use, therefore not routinely used in clinical practice. Debridement is therefore guided largely by visual assessment and physical measurements rather than objective biomechanical data and neglect the influence of plantar shear.

While increased plantar pressure has been long associated with DFU formation [11, 12], peak pressure alone is an unreliable predictor [12, 13]. Veves et al. found that only 38% of ulcers were located at peak pressure sites, with some occurring at pressures considered ‘normal’ [11, 14]. Others report ulceration occurring at both peak pressure and peak shear sites, though the dominant mechanical force remained unclear [15], and a recent systematic review reported elevated shear stress levels in patients with existing or prior DFUs compared to un‐ulcerated individuals [16].

Despite DFU formation being associated with plantar pressure and shear, the availability of measurement devices capable of capturing both components of plantar load simultaneously are limited due to the technical difficulty involved. However, research teams have begun to address this gap. Custom platforms with embedded sensors have been developed, and can capture both plantar pressure and shear, with this being at the expense of capturing data strictly under barefoot conditions [17, 18]. While in‐shoe devices have been reported that measure both components of plantar load using sensors adhered to the plantar surface or imbedded in an insole [19, 20, 21, 22, 23]. Machine‐learning techniques further enhance such measurements, enabling prediction of tissue deformation, tissue injury risk and abnormalities occurring during gait [24, 25, 26]. Although the described sensor‐based insoles offer data capture that is real‐time and reflect in‐shoe walking dynamics, the spatial resolution is constrained by the number and positioning of the sensors integrated.

To address these limitations, the University of Leeds developed the STrain Analysis and Mapping of the Plantar Surface (STAMPS) in‐shoe measurement tool, which uses a plastically deformable insole and Digital Image Correlation (DIC) to capture cumulative plantar strain during gait [27]. Measures of plantar strain reflects the combined mechanical effect of both vertical pressure and shear components of plantar load, providing insight into how the plantar surface of the foot deforms under load, with a spatial resolution of ca. 100 points per cm^2^. The STAMPS and STAMPS3D systems use this principle by quantifying cumulative strain across 10 steps through DIC, enabling assessment of combined loading even in the absence of direct shear measurement. This offers a novel approach to in‐shoe strain mapping in addition to enabling increased adaptability to a range of foot sizes and shapes, compared to other devices set to specific sizes. Technical development and validation of the system are reported in previous work [27, 28]. STAMPS provides a measure that reflects the plantar pressure and shear stresses acting across the foot by capturing their combined effect. Previous work validated the 2D system and identified ‘normal’ strain measures within a healthy cohort (*n* = 18) [29, 30], however the 2D approach is limited to planar surfaces. STAMPS3D extends this to contoured surfaces using 3D DIC, which has been demonstrated in a proof‐of‐concept study involving custom orthoses varying in stiffness [28, 31].

This paper presents a proof‐of‐concept study to evaluate whether STAMPS3D can detect localised and global strain changes in the presence of a simulated plantar callus. By introducing anatomically placed silicone inserts replicating key tactile and mechanical features of callus, this study aims to characterise how these localised changes in stiffness and surface roughness are captured by the STAMPS3D measurement system. These findings will inform future applications in diabetic populations, where subjective offloading strategies and elevated, but undetected, shear pose significant risks for DFU formation.

## 2 Methods

A proof‐of‐concept study consisting of five healthy participants was conducted. Ethical approval was obtained from the University of Leeds Ethics Committee to conduct this study (EPS FREC‐2024 1462‐2517). Prior to the walking assessments, participants were given participant information sheets and asked for written consent. Participants were included if they were > 18 years old and able to walk unaided for 50 m. Participants were excluded if they had a diabetes diagnosis, any diabetes‐related foot issues (e.g., peripheral neuropathy, peripheral vascular disease, inflammatory arthritis), plantar callus associated with pain and/or underwent regular debridement of plantar callus.

### 2.1 Simulated Callus Development

**FIGURE 1:**
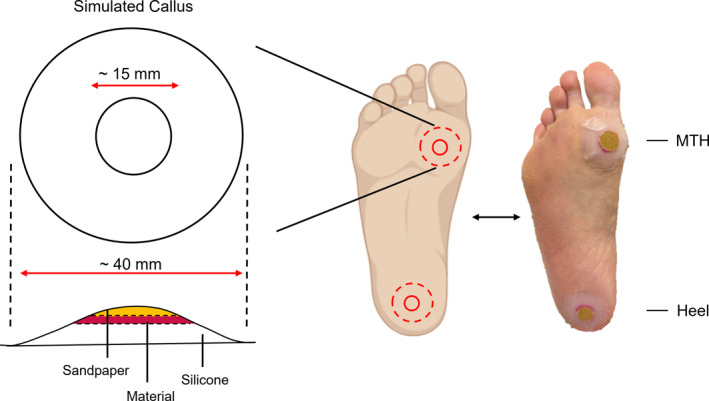
Schematic illustrating components of the multi‐layer silicone callus samples, adhered to the MTH and heel regions of participants feet.

To replicate the biomechanical effects of plantar callus, multi‐layer silicone samples were fabricated and applied to the heel and the 1st metatarsal head (MTH). A 3D printed mould with a 40 mm diameter indent and maximum central depth of 2 mm was used to cast the silicone samples. This geometry was selected to produce a gradual taper at the sample edge, ensuring a smooth integration to the surrounding plantar skin and to minimise gait disruption. Silicone (Ecoflex 00‐30, Smooth‐On Inc.) was chosen for final testing based on its values on the Shore OO hardness scale, which aligns with reported values for plantar callus at both the MTH and heel [32]. Some literature reports characteristics of plantar callus such as thickness, hardness, elasticity, hydration and surface texture compared to non‐callus sites [32, 33]. However, due to limited quantitative data on the mechanical properties of plantar callus, a representative yet simplified model was developed in consultation with experienced clinicians to encompass the most relevant tactile and structural features. The samples were cured in stages to embed two features; an embedded sandpaper layer (Grit 120) at the surface provided increased roughness (emulating coarse skin layer of callus) and beneath this a layer of woven fabric to provide localised reduction in shear strain within the callus in contrast to surrounding tissue (Figure 1). A 15 mm diameter piece of 120 grit sandpaper was first adhered to the base of the mould indent to ensure it remained at the surface of the sample. A small volume of silicone was poured into the mould, and a second material insert was placed directly under the sandpaper layer to maintain its proximity to the surface. Once cured, the mould was filled to the top and levelled to create a smooth contact interface. These features introduced a surface texture and an isolated region with altered shear behaviour, consistent with the mechanical characteristics of plantar callus.

### 2.2 Experimental Protocol

Participant shoe size was measured to size STAMPS insoles and corresponding neoprene boots (Ninewells Boot, Chaneco). Details of the STAMPS3D insole fabrication, DIC methods and analysis are reported in previous work [27, 28]. Silicone samples were adhered to the 1st MTH and heel regions of the foot using double‐sided tape, as shown in Figure 1. These regions were selected due to their clinical relevance in callus formation, with the 1st MTH also being a common site for DFUs [34, 35]. Thin knee‐high nylon socks were used to provide a consistent plantar interface.

A pre‐walk image was captured of the STAMPS insole prior to use. The STAMPS insole was then inserted into the right neoprene boot, with a similarly sized insole inserted into the left boot to prevent any differences in insole depth. Participants were asked to walk at a self‐selected walking pace for 10 steps on the right foot. This approach was adopted to promote natural gait behaviour, with consistency maintained between participants by standardising the number of steps rather than controlling walking speed. Following the walk, the insole was removed carefully, and a post‐walk image was captured of the worn STAMPS insole. This procedure was repeated with and without (control) the simulated callus adhered to each region of the participant's foot, with conditions repeated three times to account for any variability. Subsequent walking assessments were conducted using the Pedar (PEDAR INC., Novel GmbH, Munich, Germany) in‐shoe plantar pressure measurement system under both conditions. It is important to note that the STAMPS3D system captures cumulative strain, which reflects the combined effects of vertical pressure and shear stresses, whereas the Pedar system measures only vertical plantar pressure. This enabled comparison between strain and pressure distributions.

**FIGURE 2:**
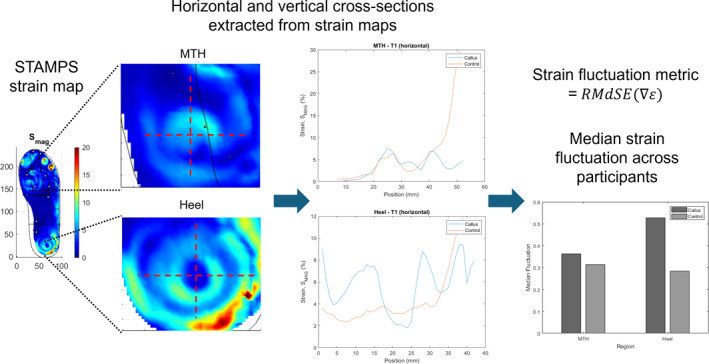
Process used to calculate the median strain fluctuation metric across participants for each region and test condition. The x‐axis on the cross‐section plots represents the distance along the extracted cross‐section and is shown at the same scale as the strain maps.

The images captured were processed using DuoDIC, a 3D DIC software, following procedures outlined in previous work [28]. Peak magnitude of strain (S_MAG_) was defined as the maximum strain value observed within each region of interest. This measure was used to identify differences between callus and control conditions across anatomical regions and whether the presence of callus has a global effect on the foot. To quantify localised variation in strain patterns, horizontal and vertical cross‐sections were extracted through the centre of the callus samples and corresponding regions in control samples. A strain‐fluctuation metric was calculated as the Root Median Squared Error (RMdSE) of the differentiated strain values along each cross‐section, using median to minimise the influence of outliers. The process of determining the strain‐fluctuation metric is demonstrated in Figure 2. Peak plantar pressure (PPP) measurements were also analysed to evaluate potential correlations between pressure and strain, and to determine whether peak pressure regions aligned with the simulated callus sites.

### 2.3 Post‐Processing and Statistical Analysis

Descriptive statistics were used to summarise strain and pressure data, with median values and interquartile ranges reported to capture central tendency and variability across participants. Paired t tests were performed for each participant and repeated to compare strain fluctuation between callus and control conditions separately for MTH and heel regions. These analyses were intended to illustrate consistency and variability across individuals, rather than to provide population level inference. Statistical significance was defined as *p* < 0.05.

PPP for each anatomical region and globally were extracted using a software mask (Novel, GmbH, Munich, Germany). Spearman's correlation coefficient was used to assess the relationship between peak S_MAG_ and PPP for both test conditions, with a significant relationship being where a moderate and statistically significant correlation was found (0.4 < rho < 0.69 and *p* < 0.05). This approach was consistent with the healthy cross‐sectional study using STAMPS [29].

## 3 Results

Five healthy participants were recruited and successfully completed the walking conditions.

**FIGURE 3:**
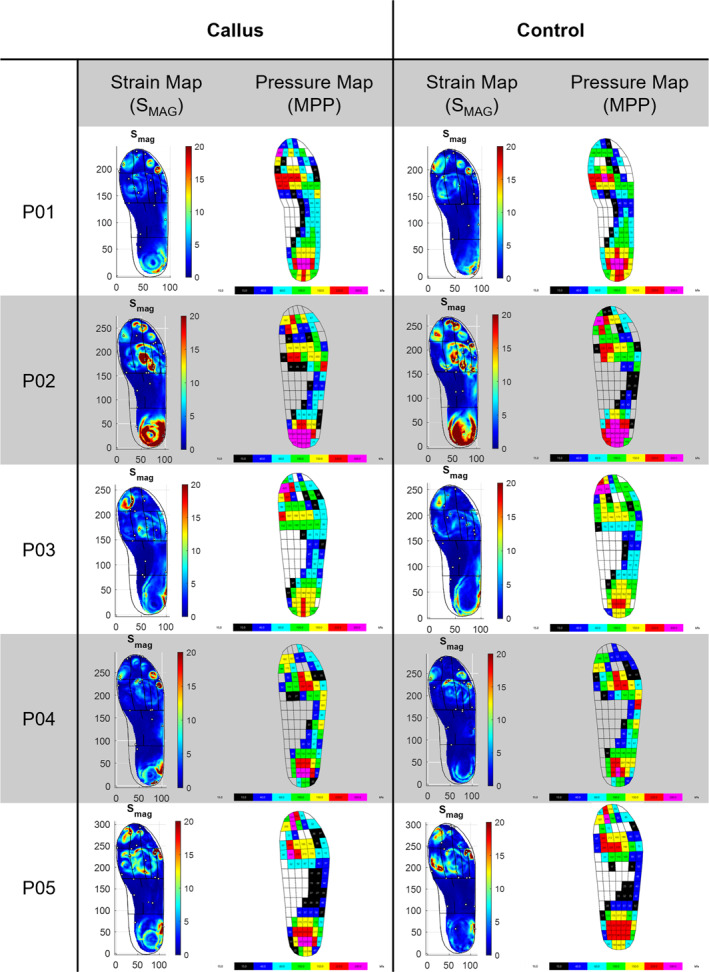
Representative examples of plantar strain and pressure distribution maps for both callus and control conditions across five healthy participants.

Representative examples of the strain and pressure maps for each condition are shown in Figure 3. While numerical differences between callus and control conditions were less clear, visual inspection revealed localised regions of changing strain in the callus condition corresponding to sample placement. These localised strain differences seen between the two conditions were particularly noticeable at the heel, where changes in strain pattern correspond to components of the callus sample. This could also be seen at the 1st MTH, however appears to be less distinct. While similar localised effects were seen in the pressure maps, visual differences between conditions were more difficult to distinguish, with increased pressure found at the forefoot and heel in both conditions. These maps illustrate the enhanced spatial resolution of STAMPS3D compared to PPP, with strain maps highlighting deformation patterns that do not always align with peak pressure regions.

Across participants, peak S_MAG_ and PPP were often found at the same anatomical regions for both callus and control conditions, with this occurring at the Hallux, 5th MTH and heel. For example, peak S_MAG_ was found at the heel in 3/5 participants and at the 5th MTH in 2/5 participants in both conditions. PPP was also found at the heel in 3/5 participants and the hallux in 1/5 participants for both conditions. PPP was found at different locations for each condition in one participant with this being at the heel and hallux/2nd toe in the callus and control conditions respectively. PPP and peak S_MAG_ were found at the same location in 2/5 participants, with this being at the heel. Measures reported did not consistently align with the simulated callus sites, reflecting either anatomical variation or edge effects. These differences are summarised in Table 1, which combines peak S_MAG_ and PPP measures across anatomical regions, reported as a median across all participants and corresponding interquartile range (IQR).

**TABLE 1:** Peak S_MAG_ and PPP reported for each condition and anatomical region of the foot, with median and IQR used to summarised data across all five participants.

	Peak S_MAG_ (%)		PPP (kPa)	
Anatomical region	Callus	Control	Callus	Control
Global	141.54 (40.22–304.18)	67.71 (59.98–361.23)	416.00 (376.50–420.50)	429.50 (328.00–462.00)
Hallux	21.29 (15.28–22.16)	15.26 (14.61–21.55)	280.00 (194.00–349.50)	333.00 (213.75–429.50)
2nd toe	18.44 (11.80–18.47)	19.59 (11.07–22.96)	265.75 (157.75–272.50)	258.75 (108.25–306.00)
Toes 3–5	23.73 (12.71–24.02)	28.97 (11.80–34.26)	144.75 (83.50–161.00)	120.25 (72.50–121.25)
1st MTH	11.07 (10.63–12.25)	15.91 (10.34–18.55)	230.50 (114.50–253.25)	154.50 (121.00–250.25)
2nd MTH	15.04 (13.60–44.91)	24.46 (13.82–35.85)	219.00 (185.00–230.50)	236.25 (183.25–250.75)
3rd MTH	7.98 (7.00–21.12)	13.70 (8.21–14.89)	172.50 (169.25–174.25)	167.00 (166.25–191.00)
4th MTH	13.02 (11.23–90.70)	18.57 (13.09–26.72)	202.25 (174.25–224.75)	190.00 (167.00–240.50)
5th MTH	11.47 (9.94–127.98)	52.08 (13.78–54.98)	203.50 (94.00–238.00)	136.25 (126.25–239.25)
Medial midfoot	1.78 (1.46–1.95)	1.78 (1.72–2.42)	13.00 (11.50–43.25)	25.00 (12.25–50.75)
Lateral midfoot	6.80 (6.20–7.91)	10.05 (9.51–12.45)	94.75 (85.25–156.75)	93.75 (60.00–148.00)
Heel	42.11 (34.25–92.23)	38.69 (32.18–59.98)	376.5 (319.25–416)	302.00 (286.50–317.25)

For the summary metrics across participants shown in Table 1, peak S_MAG_ values were higher in the callus condition across the hallux (21.2 vs. 15.3) and heel (42.1 vs. 38.7) regions, with the remaining toes and forefoot region showing elevated peak S_MAG_ in the control condition. PPP values generally showed greater pressure across anatomical regions in the callus condition, with the most prominent differences found at the toes 3–5 (144.8 vs. 120.3), 1st MTH (230.5 vs. 154.5), 5th MTH (203.5 vs. 136.3) and heel (376.5 vs. 302). From the summarised data peak S_MAG_ and PPP were found at different locations for each condition, with peak S_MAG_ and PPP both found at the heel in the callus condition and at the 5th MTH and hallux respectively in the control condition.

**FIGURE 4:**
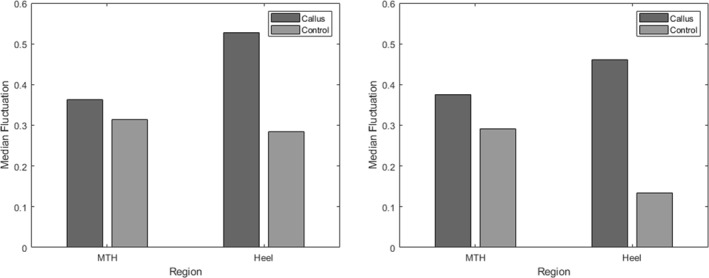
A summary of median strain fluctuation measured across horizontal (left) and vertical (right) cross‐sections across the MTH and heel regions for both callus and control conditions.

Figure 4 summarises the median strain fluctuation across participants. Fluctuation analysis revealed consistent increases in localised strain variability within the callus condition, particularly at the heel. Vertical cross‐sections through the heel region showed significantly greater fluctuation (*p* < 0.005) in all five participants, while horizontal cross‐sections showed significant increases (*p* < 0.05) in three participants. In contrast, the MTH region demonstrated less consistent differences, with only two participants showing significant increases in vertical cross‐sections and none in horizontal. These results concur with visual inspection of the strain maps, where samples at the heel produced more distinct and localised deformation patterns than those at the MTH.

**FIGURE 5:**
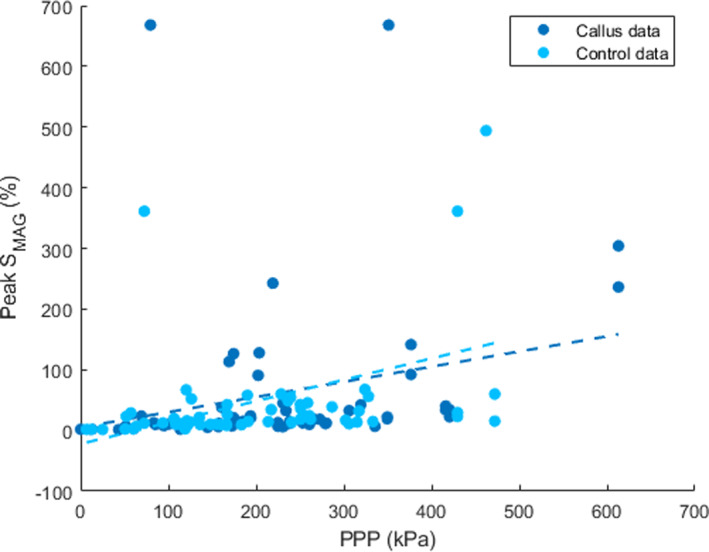
Scatter plot demonstrating the correlation between peak S_MAG_ and PPP across all participants for callus and control conditions.

Peak S_MAG_ and PPP were found to be moderately correlated for both conditions, with PPP increasing with increased S_MAG_. Spearman's correlation coefficient was found to be 0.57, *p* < 0.001 for the callus condition and 0.55, *p* < 0.001 for the control. The relationship between the two plantar measures is demonstrated in Figure 5. For each participant there was moderate to strong correlation between peak S_MAG_ and PPP for all five participants across both conditions, ranging from 0.42 to 0.76.

## 4 Discussion

This proof‐of‐concept study demonstrates that STAMPS3D can quantify local and global plantar strain patterns in the presence of simulated callus, complementing traditional pressure measurement systems. Localised strain differences were consistently found at callus sites, particularly at the heel, whereas effects at the 1st MTH were less distinct. The control conditions are similar, suggesting the effects of MTH anatomy may dominate over those of callus. The effect in pressure distribution was less pronounced but showed similar trends, consistent with typical loading patterns of walking [36, 37]. The reduced sensitivity in pressure measures may reflect the reduced spatial resolution compared to STAMPS3D, (ca. 8 vs. 560 datapoints at 1st MTH respectively). These findings highlight the enhanced spatial resolution of strain‐based metrics compared to pressure alone, enabling STAMPS3D to capture subtle, anatomically specific changes in plantar loading.

Across participants, peak S_MAG_ tended to increase at the heel and hallux with callus; regions typically experiencing high loads during gait and linked to ulceration [35, 36, 37, 38]. Since heel loading is largely unavoidable, this increased strain is consistent with heel‐strike mechanics. Forefoot regions were varied, with some responses showing higher S_MAG_ without callus. Lower forefoot strain under the callus condition may reflect an individual's anatomical structures influencing load distribution and (in this cohort of healthy individuals with intact sensation) subconscious gait adaptation to offload areas where a foreign object is perceived. In this proof‐of‐concept study, anatomical foot shape was not recorded, but these results indicate that this should be included in future studies. PPP measures were elevated in 8/11 regions under the callus condition, commensurate with reports of elevated pressure at callus sites in diabetic cohorts (particularly forefoot and heel) with reduction (25%–30%) following debridement, increased to 54% when combined with orthoses [7, 8]. Healthy cohorts also show higher pressure with callus, but debridement does not reduce this, highlighting differences in tissue resilience and adaptation [3]. The larger global peak S_MAG_ observed in the callus condition may partly reflect inter‐participant variability, as one participant exhibited substantially higher strain values at the heel in both conditions, influencing the overall median. In addition, STAMPS3D identified regions of elevated strain outside the simulated callus sites, features not detected by the Pedar system, which measures only vertical pressure. This likely contributes to the greater global difference in strain compared with PPP. It is also possible that the short walking protocol (10 steps) captured only the early response to altered local stiffness, where more pronounced differences around the callus regions may emerge over longer cumulative loading. Alternative measures such as median strain across each anatomical region were also considered to capture cumulative deformation effects. These showed similar trends but were less directly comparable to the PPP data, which reflect maximum vertical loading. For consistency in benchmarking between systems, peak S_MAG_ was used as the primary measure, though future work may explore median or cumulative strain metrics to assess broader intra‐regional strain behaviour.

Peak S_MAG_ and PPP occurred at the heel under the callus condition, but their locations differed in the control condition. This suggests that shear strain measures may capture aspects of local tissue deformation not reflected in pressure alone. This hypothesis is consistent with studies showing elevated peak‐to‐peak shear at callus sites despite non‐significant PPP differences [10]. Callus is a common precursor to ulceration in people with diabetes and peripheral neuropathy, where altered plantar loading and reduced protective sensation increases risk [2]. In contrast, healthy individuals may also develop callus, but typically without the same pathological consequences, reflecting differences in tissue resilience and mechanical adaptation. Our results show that strain variability can be detected even in non‐neuropathic feet, supporting the notion that STAMPS3D may help distinguish adaptive and maladaptive callus in diabetic cohorts. Correlations were moderate and significant between PPP and peak S_MAG_ in both conditions (rho = 0.57 and rho = 0.55, *p* < 0.001), aligning with prior findings from a healthy cohort (rho = 0.62, *p* < 0.001) [29]. A moderate correlation was expected, as peak S_MAG_ reflects cumulative deformation arising from both vertical pressure and shear loading, whereas PPP represents only the vertical component. The two measures are therefore related but not interchangeable. The strength of the correlation observed here and consistency with previous findings in healthy cohorts, suggests that while pressure contributes substantially to overall strain, STAMPS3D also captures additional shear‐related deformation that is not represented in PPP alone. Beyond peak values, a strain variability metric was developed to provide a novel means to assess complex strain responses. Strain variability increased consistently under callus, particularly at the heel, concurring with visual differences in strain maps. This metric may help capture subtle load changes not evident from peak shear and may provide insight into why regions such as the MTH and heel are more prone to tissue breakdown in neuropathic feet. These local strain‐fluctuation patterns further demonstrate the sensitivity of STAMPS3D to detect subtle variations in plantar loading that are not captured by vertical pressure systems. The high spatial resolution of the strain maps enables identification of localised deformation responses at the callus sites that remain invisible to pressure‐only devices such as Pedar. This level of measurement resolution is beyond the capability of current in‐shoe systems and highlights the potential of strain‐based metrics to reveal clinically relevant loading behaviour that may otherwise go undetected.

Beyond the immediate findings, this work builds on a previous STAMPS3D study demonstrating the system's ability to quantify plantar load on contoured, variable stiffness orthoses, reinforcing its potential relevance to clinical offloading practice [31]. In contrast, the present study was focused on using the flat STAMPS insole, with no orthotic device, to isolate the effect of the simulated callus without interactions from contoured geometry. Offloading and debridement are the two primary management strategies recommended by the IWGDF for preventing and treating DFUs [1], yet current clinical decision making relies heavily on visual inspection and pressure‐only metrics, which do not capture shear‐related tissue deformation. By providing a cumulative, spatially detailed measure of plantar strain, STAMPS3D offers a practical, low‐cost and potentially feasible for bedside implementation as an alternative to laboratory‐based plantar load measurement systems. This positions STAMPS3D as a promising tool for objective assessment of callus‐related loading, evaluation of debridement effectiveness and optimisation of offloading interventions in people with diabetes.

Limitations of the study relate primarily to the small proof‐of‐concept sample size, limiting the ability to generalise, therefore results should be interpreted with caution. The STAMPS3D technique is inherently a compound measure, thus precluding capture of dynamic data, but bringing increased spatial resolution compared to real‐time sensor systems [28, 31]. The simulated callus may not fully replicate the complex mechanical properties of plantar callus in vivo but does recreate local variation in tissue stiffness reported in literature. Future work will evaluate STAMPS3D for clinical use, focusing on people with diabetes and neuropathy, to assess whether strain metrics can identify callus‐associated deformation and DFU risk. Longitudinal studies could reveal high‐risk regions and evaluate whether STAMPS3D can guide debridement, individual susceptibility to ulceration and personalised off‐loading approaches.

## 5 Conclusions

This proof‐of‐concept study in healthy participants demonstrates that STAMPS3D can detect localised differences in plantar strain associated with simulated callus, complementing pressure‐based measures. While pressure metrics showed modest differences between callus and control conditions, strain measures revealed consistent, anatomically specific changes, particularly at the heel. These findings highlight the virtue of strain‐based metrics in capturing tissue‐level deformation that may be indistinct in pressure alone. The study establishes methodological feasibility and provides a foundation for future clinical evaluation. STAMPS3D can help bridge the gap between pressure‐based monitoring and tissue‐level biomechanics to support risk assessment and intervention planning; guiding debridement strategies and offering new insights into ulceration risk and prevention. Given its low‐cost, adaptable design and minimal technical requirements, STAMPS3D has the potential to evolve into a clinically practical, bedside tool aligned with IWGDF offloading recommendations.

## Author Contributions

**Francesca Sairally:** visualization, data curation, formal analysis, writing – original draft, writing – review and editing, methodology, conceptualization, project administration, investigation. **David A. Russell:** supervision, conceptualization, writing – review and editing, methodology. **Heidi J. Siddle:** supervision, conceptualization, writing – review and editing, methodology. **Daniele Trinca:** writing – review and editing, resources. **Claire Brockett:** supervision, conceptualization, writing – review and editing, methodology. **Peter R. Culmer:** supervision, conceptualization, writing – review and editing, methodology.

## Funding

This work was supported by the United Kingdom Engineering and Physical Sciences Research Council (EPSRC) grant for the University of Leeds' Doctoral Training Partnerships (2022/23). This research project was also supported by the NIHR Leeds Biomedical Research Centre (IS‐BRC‐1215‐20015). The views expressed in this publication are those of the author(s) and not necessarily those of the NIHR, NHS, or the Department of Health and Social Care. David Russell is supported in part by the NIHR Advanced Fellowship (NIHR300633).

## Ethics Statement

All relevant ethical guidelines followed and The Faculty Research Ethics Committee for Engineering and Physical Sciences of the University of Leeds gave ethical approval for this work.

## Consent

Informed written consent obtained from the participant of the case study.

## Conflicts of Interest

The authors declare no conflicts of interest.

## Data Availability

The datasets generated for this study can be found in the Research Data Leeds Repository (https://doi.org/10.5518/1820).
